# Growth performance, carcass characteristics and cost implications of supplementing Turkey poults with toasted Bambara nut by-products

**DOI:** 10.1016/j.vas.2022.100250

**Published:** 2022-04-04

**Authors:** Kingsley C. Okonkwo, Bennett E. Obua, Ugochukwu B. Ifenkwe, Aduli E.O. Malau-Aduli

**Affiliations:** a Department of Animal Science and Animal Production, Michael Okpara University of Agriculture, Umudike, Abia State, Nigeria; b Animal Genetics and Nutrition, Veterinary Sciences Discipline, College of Public Health, Medical and Veterinary Sciences, Division of Tropical Health and Medicine, James Cook University, Townsville, Queensland 4811, Australia

**Keywords:** Turkey poults, Bambara nuts, Carcass characteristics, Economic analysis, Supplementation

## Abstract

Legumes provide high quality proteins with nutritional benefits for monogastric animals. Compared to other legume pulses, Bambara nuts (*Vigna subterranean*) grow in marginal soils, contain 12 different proteins and 214 identifiable peptides that readily provide nutritional and health benefits to consumers. Toasted Bambara nuts are consumed by humans, while the shells are ground and included in both poultry and ruminant rations as a readily degradable protein source. The hypothesis that turkey poults supplemented with toasted Bambara nuts by-products will respond with higher daily weight gains, faster growth, superior carcass characteristics, heavier organs and higher profit margins than unsupplemented poults was tested in this study with the primary objective of evaluating the response of grower turkey poults to supplementation with graded levels of Bambara nut by-products. The feeding trial lasted for 56 days and a completely randomised experimental design was utilised to randomly allocate 60 poults to five treatments of 12 poults each, comprising the control (0%), 7.5, 15, 22.5 and 30% inclusion level of toasted Bambara nut by-products. Compared to the control group, supplemented poults achieved a significantly better (*P* < 0.05) feed conversion ratio, heavier carcass traits, total and eviscerated organ weights and faster average daily gains. This superior performance was highest in poults supplemented at the 30% inclusion level which returned an 85% profit margin and a 10% reduction in feed cost compared to unsupplemented poults. These findings imply that turkey farmers can break even while achieving superior growth performance, heavier carcasses and nutritional benefits accruing from supplementation with toasted Bambara nuts by-products, hence the need to accept the tested hypothesis.

## Highlights

• Higher feed conversion ratio, average daily and total weight gains achieved in supplemented poults at 30% inclusion level of Bambara nut by-products.
• Poults supplemented at 30% inclusion level of Bambara nut by-products had higher carcass and organ weights.
• A 10% reduction in cost and 85% profit margins achieved at 30% inclusion level of Bambara nut by-products.

## 1 Introduction

The increase in global demand for high quality meat and escalating costs of production due to scarcity of feed resources have necessitated the search for alternative poultry ration formulation ingredients (Mota de Carvalho, Oliveira, Saleh, Pintado & Madureira, 2021). Feed costs account for more than 65% of overall production cost (Moss, Chrystal, Crowley & Pesti, 2021a), and due to the high cost of poultry feeds, there is continuous pressure on farmers to be innovative in formulating 'least cost' diets that are not only affordable, but also able to meet the nutritional requirements of animals (Alhotan, 2021; Moss, Chrystal, Crowley & Pesti, 2021b). Drought, poor agricultural practices and plagues have also led to unviable farm harvests, especially in third world countries. Padilla, Liverpool-Tasie and Myers (2021) explored the decision-making process of commercial poultry enterprises facing rising input costs in Nigeria. They reported that while Nigeria has seen a rapid growth of commercial livestock enterprises, the sensitivity of the poultry industry to changes in feed prices is a major threat to the sector. These fluctuations in feed prices tend to be exacerbated by the existing competition for feedstuff between humans and livestock, thus increasing the cost of conventional animal feedstuff and consequently, the cost of poultry products (Truong et al., 2021; Vladimirovna et al., 2020).

In a bid to solve the challenges of feed cost and shortages, several locally available alternatives such as by-products from crop harvests, processing, biomass, and foliage have been explored (Alencar et al., 2021; Dida, Challi & Gangasahay, 2019; Li, Zhou, Xu & Zi, 2019; Pandi et al., 2016). The partial replacement of conventional livestock feeds with these by-products is common in developing countries such as Nigeria, and has been reported in previous research (Ashayerizadeh, Dastar, Shargh, Mahoonak & Zerehdaran, 2018; Damasceno et al., 2020; Souza et al., 2020). Bambara nut by-products have been successfully used as additives/supplements in fingerling, rabbit and broiler feeds (Nnamani, 2010). Unlike other additives such as groundnuts, soybeans, and cowpeas, Bambara nut by-products are readily degradable sources of both dietary energy and protein Onyimonyi and Ugwu (2007); Ukpabi, Amaefule and Amaefule (2008). They are economically viable feed supplements for pigs (Okah, Ubochi & Uzoma, 2016; Onyimonyi & Okeke, 2007), egg layers, broilers and spent layers (Onyimonyi & Ugwu, 2007; Ugwu & Onyimonyi, 2008).

Although cheap and easily sourced, low-cost feed supplements such as Bambara nut by-products, contain secondary metabolites, alkaloids and tannins (Diarra, 2018; Woyengo, Beltranena & Zijlstra, 2017) that reduce the nutritional quality and efficiency of raising monogastric animals. It is essential to minimise the impact of these anti-nutritional factors using heat treatment-based approaches prior to utilisation for animal feeding. Toasting effectively reduces the impact of metabolic inhibitors and enhances amino acid and fibre utilisation (Okomoda, Tiamiyu & Akpan, 2017). Obua, Okocha, Ekereuke and Mbenyi (2017) showed that replacing the feed of starter turkey birds with toasted Bambara nut by-products up to 22.5% dietary inclusion level does not negatively impact growth performance and production cost. However, published reports on its performance when used as a partial feed replacement for grower turkey poults, impact on carcass characteristics and cost implications are scarce. Therefore, in the current study, the effect of toasted Bambara nut by-products on the growth performance, organ and carcass characteristics of grower turkey poults was investigated to fill this existing knowledge gap in monogastric nutrition. Furthermore, the cost-benefit analysis and economic implications of including toasted Bambara nut by-products in the feeds of turkey poults were also assessed. We tested the hypothesis that turkey poults supplemented with toasted Bambara nut by-products will have higher daily weight gains, faster growth, superior carcass characteristics, heavier organs and higher profit margins than unsupplemented poults. The primary objective was to evaluate the profitability and response of grower turkey poults to supplementation with graded levels of Bambara nut by-products and the resultant effect on growth and carcass characteristics.

## 2 Materials and methods

### 2.1 Animal ethics, location and experimental design

All procedures and animals utilised in this study were carried out at the Poultry Unit of the Teaching and Research Farm of Michael Okpara University of Agriculture, Umudike, Nigeria, in compliance with institutional animal ethics approved guidelines under internationally acceptable best practice welfare standards for the care and use of animals for scientific purposes. Prior to allocation into treatment groups, the experimental turkey poults were acquired at day-old and brooded for three weeks on a high-protein ration (30% CP). From the 4th to the 8th week, a starter ration whose composition is shown in Table 1 was introduced. Routine vaccination regimes against Newcastle disease, fowl pox, infectious bursal disease and coccidiosis were strictly adhered to. At 8 weeks of age, the poults were offered an experimental ration (Table 2) and a completely randomised experimental design was used to randomly allocate sixty turkey poults balanced by weight, into five treatments of 12 animals each, on the basis of the following percentage inclusion levels of toasted Bambara nut by-products: T_1_ (Control, 0%), T_2_ (7.5%), T_3_ (15%), T_4_ (22.5%) and T_5_ (30%). The feeding trial lasted for 56 days including a 14-day adaptation period.

**Table 1:** Ingredient composition of the starter feed.

Ingredients	Composition (%)
Maize	43.65
Soya bean meal	23.00
Groundnut cake	20.00
Foreign fish meal	9.00
Bone meal	3.00
Salt	0.25
Vitamin/mineral premix	0.50
Lysine	0.30
Methionine	0.30
**Total**	100

**Table 2:** Feed ingredient composition of the experimental diet.

Ingredient composition (%)	T_1_	T_2_	T_3_	T_4_	T_5_
Maize	57.85	52.35	46.85	41.35	35.85
Soy bean meal	15.00	15.00	15.00	15.00	15.00
Groundnut meal	18.00	16.00	14.00	12.00	10.00
Fish meal	5.00	5.00	5.00	5.00	5.00
Toasted Bambara nut by-products	0.00	7.50	15.00	22.50	30.00
Methionine	0.30	0.30	0.30	0.30	0.30
Lysine	0.30	0.30	0.30	0.30	0.30
Salt	0.25	0.25	0.25	0.25	0.25
Vitamin/mineral premix	0.30	0.30	0.30	0.30	0.30
Bone meal	3.00	3.00	3.00	3.00	3.00
**Total**	100	100	100	100	100
**Calculated composition**					
Crude protein (%)	22.04	22.12	22.22	22.30	22.40
ME (kcal/kg)	2986.70	2969.90	2911.90	2854.10	2816.40

### 2.2 Feed procurement, processing and proximate analysis

Feed ingredients and raw Bambara nut by-products (BGO) were procured locally from farms and processing mills in Umuahia, Abia State, Nigeria. Toasting was carried out for 15 min using the procedures described in detail by Obua et al. (2017). Proximate analysis of the toasted Bambara nut by-products showed that it contained 88.68% dry matter, 18.52% crude protein, 4.74% crude fibre, 4.47% ether extract, 4.83% ash, and 56.17% nitrogen free extract (Obua et al., 2017).

### 2.3 Evaluation of growth performance and carcass characteristics

At 16 weeks of age, randomly selected poults representative of each treatment, were fasted overnight, before being humanely sacrificed in accordance with poultry processing standards. Measurements of initial and final live weights, pre-fasting and slaughter weights and carcass dressed weights were taken using an electronic weighing balance (error = ±0.001 g) for evaluating growth performance, carcass and organ characteristics.

The following computations were used to evaluate growth performance:

(1) Totalbodyweightgain=Finalbodyweight−Initialbodyweight

(2) $$Average\;daily\;feed\;intake\;\left(g\right)=\;\frac{Quantity\;of\;feed\;offered\;-\;Residuals}{No.\;\;of\;birds\times No.\;\;of\;days\;of\;the\;experiment}$$

(3) $$Average\;daily\;weight\;gain\;\left(g\right)=\;\frac{Final\;\;live\;weight\;-Initial\;weight}{No.\;\;of\;birds\;\times No.\;\;of\;days\;of\;the\;experiment}$$

(4) $$Feed\;conversion\;ratio\;\left(FCR\right)=\frac{Quantity\;of\;feed\;consumed}{Weight\;gained}$$

(5) $$Mortality\;\;\left(\%\right)=\frac{No.\;\;of\;dead\;birds}{Initial\;stock\;at\;the\;beginning\;of\;the\;experiment}\times 100$$

Dressing percentage, carcass and eviscerated weights of the thighs, wings, breasts, back cuts, drumsticks, shanks, kidney, spleen, liver, heart, gizzard and proventriculus were evaluated using Eqs. (6) – 8.

(6) $$Dressing\;percentage\;\left(\%\right)=\frac{Carcass\;weight}{Live\;weight}\times 100$$

(7) $$Carcass\;cut\;part\;\left(\%\right)=\frac{weight\;of\;carcass\;cut\;part}{live\;weight}\times 100$$

(8) $$Organ\;proportion\;\left(\%\right)=\frac{weight\;of\;organ}{live\;weight}\times 100$$

### 2.4 Economics of production

The economics of raising grower turkey poults using toasted Bambara nut by-products was evaluated using the market cost of feed ingredients and price per kilogram of turkey meat in Nigeria as at the time of this experiment (June 2017). The exchange rate as at June 2017 was 1USD = ₦318.32. Parameters considered were cost of formulating 100 kg of feed (₦), cost of feed consumed per kilogram (₦), cost per kilogram weight gain, revenue (₦) and gross margin (₦).

### 2.5 Statistical analysis

Data were analysed using the Statistical Analysis System software version 9.4 (SAS Institute, Cary, NC, USA). Initial data screening was carried out by computing summary statistics of means, standard deviations, minimum and maximum values to examine data for entry errors and outliers. Data were analysed by linear mixed model (PROC MIXED) procedure with the fixed effect of treatment (0, 7.5, 15, 22.5 and 30% toasted Bambara nut by-product inclusion) and pen nested within treatment as a random effect to determine the effect of treatment on growth performance, carcass characteristics, organ proportions and economics of production. Baseline measurements of initial live weights were included as covariates in the model. When the effect of treatment was significant (*p* < 0.05), orthogonal polynomial contrasts were performed to test for linear, quadratic and cubic responses to increasing the proportions of toasted Bambara nut by-products. Significant interactions were separated using the Tukey-Kramer pairwise comparison test. The quadratic and cubic responses were eventually dropped from the model because they were not significant.

## 3 Results

### 3.1 Evaluation of growth performance

The growth performance of poults is shown in Table 3. Treatment differences in final live weight, average daily weight gain, total weight gain and feed conversion ratio (FCR) were significant (*p* < 0.05). The final live weights, total weight and average daily weight gains of all supplemented poults were higher than those of the unsupplemented control (T_1_), the highest overall total weight gained equating to a 39.4% increase. There was a linear increase in average daily weight gain as the proportion of toasted Bambara nut by-products increased in the diet with poults in T_4_ and T_5_ recording significantly higher gains than their contemporaries in T_1_, T_2_ and T_3_. Feed conversion ratio (FCR) – an indication of the ability of the animals to convert the feed to desired meat output (carcass yield) ranged from 7.71 to 5.41, with supplemented poults being more efficient converters than the unsupplemented control (T_1_). The mortality rate was negligible, not significantly different and could not be attributed to treatment effects.

**Table 3:** Growth performance of grower turkey poults in response to dietary supplementation with varying levels of toasted Bambara nut by-productsβ.

Parameters	T_1_ (0%)	T_2_ (7.5%)	T_3_ (15%)	T_4_ (22.5%)	T_5_ (30%)	SEM
Initial weight (g)	945	978	963	971	941	22.40
Final weight (g)	2190.70^C^	2499.83^b^	2452.23^b^	2677.67^ab^	2816.67^a^	63.80
TWG (g)	1245.7^c^	1521.36^bc^	1488.70^bc^	1705.83^ab^	1875.33^a^	56.48
ADWG (g/day)	24.08	27.17	26.59	30.47	33.49	0.69
Feed intake (g/day)	185.66^a^	181.50^b^	181.34^b^	181.30^b^	181.18^b^	0.21
Mortality (No)	1.0	–	–	–	–	0.14
FCR	7.71^a^	6.68^ab^	6.82^ab^	5.95^bc^	5.41^c^	0.24

β *TWG = Total weight gain; ADWG = average daily weight gain; FCR   feed conversion ratio. ^a,b,c^ means on the same row with different superscripts are significantly different (p < 0.05)*.

### 3.2 Carcass characteristics

Table 4 shows the carcass characteristics of the experimental poults. The inclusion of toasted Bambara nut by-products in the diet of led to a simultaneous increase in weight at slaughter, while eviscerated and dressed weights of the supplemented poults in T_2_, T_3_, T_4_ and T_5_ were statistically similar (*p* > 0.05). Supplemented poults had lesser feather to flesh ratio, hence a decrease in dressing percentage. Carcass components measured as percentages of live weight showed significant differences (*p* < 0.05) between the breast and neck, while the head, back cut, wing, shank, drum stick, and thigh were similar between treatment groups, hence the dressed weights of the saleable carcass components were comparable.

**Table 4:** Carcass characteristics of poults fed varying dietary levels of toasted Bambara nut by-productsβ.

Parameter	T_1_	T_2_	T_3_	T _4_	T_5_	SEM
Live weight at slaughter (g)	2133.33^b^	2583.33^a^	2433.33^ab^	2613.33^a^	2763.33^a^	73.15
Eviscerated weight (g)	1583^b^	1903.33^a^	1766.67^ab^	1916.67^a^	2007.67^a^	50.68
Dressed weight (g)	1463.33^b^	1749.67^a^	1616.67^ab^	1763.33^a^	1838.67^a^	46.91
Dressing percentage (%)	68.52^a^	67.80^ab^	66.43^b^	67.48^ab^	67.30^ab^	0.26
Carcass components (% of live weight)						
Back	11.14	11.20	10.98	11.05	10.88	0.05
Breast	16.64^a^	16.13^ab^	15.82^ab^	14.42^b^	15.48^ab^	0.31
Wing	11.30	11.35	11.18	11.19	11.02	0.05
Drum stick	11.07	11.09	10.93	10.97	10.87	0.15
Thigh	9.11	9.24	9.09	9.33	9.06	0.05
Neck	4.75^b^	4.89^ab^	4.96^ab^	5.04^a^	4.92^ab^	0.03
Shank	3.21	3.19	3.25	3.33	3.23	0.02
Head	2.96	2.95	2.90	3.03	2.98	0.018

β *T_1_ –T_5_ as in*Table 3*. ^a,b^ means in rows with superscripts are significantly different(p < 0.05). SEM = Standard error of mean*.

### 3.2 Organ proportions

Significant differences (*p* < 0.05) were observed between the proportions of abdominal fat, liver and lungs (Table 5), whereas proportions of the gizzard/proventriculus, kidney, heart, and spleen were similar between treatment groups (*p* > 0.05).

**Table 5:** Organs as proportions of live weight in poults supplemented with varying dietary levels of toasted Bambara nut by-productsβ.

Organs (% of live weight)	T_1_	T_2_	T_3_	T _4_	T_5_	SEM
Abdominal fat	0.29^c^	0.34^b^	0.35^ab^	0.36^ab^	0.38^a^	0.009
Gizzard + proventriculus	3.43	3.40	3.36	3.29	3.34	0.02
Liver	1.43^b^	1.56^a^	1.53^ab^	1.52^ab^	1.52^ab^	0.017
Kidney	0.45	0.48	0.47	0.47	0.49	0.026
Heart	0.39	0.40	0.38	0.39	0.39	0.003
Lung	0.57^b^	0.61^ab^	0.63^a^	0.62^ab^	0.59^ab^	0.008
Spleen	0.13	0.13	0.13	0.14	0.13	0.001

β *T_1_ –T_5_ as in*Table 3*. ^a,b^ means in rows with different superscript are significantly different(p < 0.05). SEM = Standard error of mean*.

### 3.3 Economics of production

Economic evaluation of the production process is shown in Table 6. There were significant differences in revenue, gross margin and cost per kilogram of weight gain between the treatment groups. Supplementing the poults with toasted Bambara nut by-products reduced the cost of production and led to higher gross margins (Table 6).

**Table 6:** Economics of producing grower turkey poults using varying levels of toasted Bambara nut by-products as feed supplement β.

Parameters	T_1_	T_2_	T_3_	T_4_	T_5_	SEM
Cost/kg of feed(₦)	190.62	185.24	180.12	175.49	170.12	8.89
Cost/kg weight gain(₦)	1469.68^a^	1237.40^b^	1228.41^b^	1044.16^c^	920.35^c^	68.08
Cost of feed consumed(₦)	1906.20	1852.40	1800.20	1754.90	1701.20	7.97
Revenue(₦)	3280.00^c^	3745.00^b^	3675.00^b^	4015.00^ab^	4225.00^a^	96.06
Gross margin(₦)	1373.80^c^	1892.60^b^	1874.80^b^	2260.10^ab^	2523.80^a^	75.07

β *T_1_ –T_5_ as in*Table 3*. ^a,b,c^ means on the same row with different superscripts are significantly different (p < 0.05)*.

## 4 Discussion

### 4.1 Growth performance

The growth performance data reinforced the ability of toasted Bambara nut by-products (TBNBP) to adequately supply readily absorbable nutrients that meet the requirements for growth without any detrimental effects on supplemented poults. This finding of heavier finishing weights in supplemented poults than their unsupplemented peers is in tandem with other previous studies (Torhemen, Agabi, Adi & Torhemen, 2020; Ukpabi et al., 2008). The higher weight gain exhibited by the poults supplemented with TBNBP is reflective of the higher intake and demonstrates that up to 30% of the diet can be substituted with TBNBP without negatively impacting daily feed intake and growth performance of the birds. A similar trend was also observed in broilers supplemented with Bambara nut by-products (Tuleun, Okereke & Sunmola, 2020). While a number of physiological and environmental factors such as temperature, feed form and flavour have been observed to affect feed conversion ratio, our results in the present study showed that birds supplemented with TBNBP were more efficient converters than their unsupplemented counterparts. This may be attributed to improved palatability, digestibility and higher metabolic activities associated with the growth phase contributing to improved feed conversion efficiency and immunity as indicated by low mortality. A combination of top quality brooding practices and favourable impact of TBNBP as a partial replacement for soybean meal in the formulated diets could possibly explain the efficient utilisation and conversion of nutrients to superior growth in supplemented poults.

### 4.2 Carcass characteristics

TBNBP highest slaughter weight observed in poults at the 30% inclusion level (T_5_) significantly translated into higher average eviscerated and dressed weights (Table 4) probably due to improved feeding value attributable to toasting as previously reported by Okomoda et al. (2017). The similar live weight at slaughter observed between all supplemented treatments contradicts the findings of Torhemen et al. (2020) and Ukpabi et al. (2008) which reported that Bambara nut by-products reduced the live weight and average weight gain of broilers. It would seem that the duration of toasting in our study optimally minimised the impact of metabolic inhibitors and prevented the denaturing of the proteins in the TBNBP, hence, the exceptional conversion of absorbed nutrients into body weight by the grower turkey poults.

The similar dressing percentages in poults could be attributable to the low concentration of cysteine, known to aid feather development, which can in turn, affect dressing percentage. In our study, the percentage of cysteine was below the recommended level by Hybrid Genetics (2016). Regardless, the poor economic value of feathers and similarity in the result obtained from measured carcass parameters are pointers to the fact that nutrient supplies from the experimental diets were adequate and produced comparable performance parameters. There was no logical explanation for the significant differences observed in breast weight with increasing TBNBP inclusion levels and the highest neck, thigh, shank and head weights in poults fed diet T_4_ (Table 4), but comparable carcass proportions support the earlier assertions that at various levels of TBNBP inclusion, there were no deleterious effects on overall performance.

### 4.3 Organ proportions

The nature and composition of feed supplements can affect the functioning of the internal organs of monogastric animals, hence the observed trend in this study where the liver of unsupplemented poults (T_1_) were different from those of their TBNBP-supplemented peers. The heavier livers in supplemented poults (T_2_, T_3_, T_4_ and T_5_) may likely be due to residual anti-nutritional factors, but might as well be a reflection of heavier live weights because organ growth is proportional to animal size. A similar observation was evident in the relatively heavier weights of the lungs and abdominal fat of supplemented poults, but the reverse was displayed in unsupplemented poults with the heaviest gizzard. This can be attributed to muscle enlargement caused by the stress of churning feeds with high dietary crude fibre which is known to affect gizzard size in monogastric animals (Tejeda & Kim, 2021). The comparable relative weight of the spleen, kidney and heart in all supplemented poults indicates that these vital organs were able to optimally perform their physiological activities due to sufficient nutrients needed to support normal organ function and development since insufficiency of readily utilisable amino acids can inhibit organ growth (Alagawany et al., 2020; Swatson, Gous, Iji & Zarrinkalam, 2002).

### 4.4 Economics of production

Reduction in overall production cost is a major goal of supplementing animals with agro-industrial by-products. As depicted in Table 6, the inclusion of TBNBP significantly reduced the costs of producing a kilogram of feed, kilogram weight gain and feed consumed. This observation followed the expected trend because TBNBP is cheaper than other feed ingredients such as maize, groundnut and sorghum. The lower feed cost per kilogram of meat produced from TBNBP-supplemented feeds suggests that TBNBP is a more economically viable and sustainable poultry feed supplement that can be incorporated up to 30% level in turkey diets. Additionally, incorporating higher quantity of TBNBP in the diets of poults improved the feed conversion ratio, hence the reduction in unit cost per weight gain. Accruable revenue and gross margins are dependant on cost of feed and feed conversion ratio. Our data clearly demonstrate that incorporating 22.5 – 30% TBNBP in the diet of grower turkey poults can reduce feed cost by 8 – 10.75% and increase gross margin by 65 – 84%.

## 5 Conclusion

This study has shown that TBNBP can be incorporated in diets of grower Turkeys without hindering growth performance, carcass and organ characteristics of supplemented poults. Feed conversion ratio was improved with increasing inclusion levels of TBNBP, indicating the effectiveness of toasting. It may be worthwhile exploring the options of pelletizing, extrusion and enzyme addition as a means of improving feed conversion ratio. It was apparent from our study that TBNBP-supplemented diets contained sufficient nutrients propelling adequate growth and development of the organs and dressed carcasses in poults. Therefore, grower poults can be supplemented with TBNBP diets up to 30% inclusion level without any negative impacts on live weight or organ function. Finally, a significant cost reduction by 8 – 10.75% and 65 – 84% increase in gross margin by can be achieved by incorporating TBNBP in the diet of grower turkeys.

## Animal ethics statement

The breeding and treatment of the birds were in accord with institutional and internationally acceptable best practice.

## Declaration of Competing Interest

The authors confirm that they have no conflict of interest.
